# Scorpion Hunting on the Karroo

**Published:** 1909-12-25

**Authors:** 


### The Practitioner's Relaxations.

### SCORPION HUNTING ON THE KARROO.

At Christmas time the Karroo is a hard-baked desert, musical with the notes of myriads of entomological treasures, the happiest hunting ground for him who takes a fond and semi-scientific interest in the life habits of the various creatures that the ignorant include under the convenient generalisation of "insects." In the mimosæ, a line of fading gold bordering the dried-up water-courses, huge cicadas screech and chirrup; the fearsome looking *korn boer*, popularly accredited with venomous qualities, though in reality it is the most placid of tree crickets, notwithstanding its spiky armour-plated panoply, struts slowly from trunk to twig; under a parched mesembryanthemum bush, whose violet flowers are faded out of all recognition of their September splendour, lies a dreamy puff adder, mimicking the motley of the iron clay nodules that environ it. Each large flat stone shelters its colony of insect and reptiledom, while in more sheltered spots, among the tufts of dry Bushman grass, a host of species await the enthusiastic collector who dares the fierce December sun and feels active enough to go hunting when the thermometer registers 116° F. in the shade. Scorpion collecting, under such conditions, is hardly a treat; but it is an occupation so absorbing in its pleasures to one who delights in Nature study, and so rich in its results that no collector can afford to let the opportunity slip. To wait till the cooler evening hours is to let many of the choicest specimens go. At sunset they leave their burrows and their sheltering stones and go hunting themselves. At mid-day they lie patiently awaiting the inquisitive collector who has the temerity to disturb their siesta.

All the collector needs are a good strong geological chisel to make an impression on the hard clay if it is desired to dig up a burrow, a large wide-mouthed collecting jar filled with methylated spirits in which to keep his captures, and a set of labels or a number of strips of tin on which to record his notes. A short walk over the nearest strip of veld will reveal plenty of flat stones to be turned over. Underneath these are to be found the various treasures, of which a selection can easily be made and consigned to the collecting jar. Here, for instance, is a likely one—a flat boulder of clay slate, marked with the arborescent magnesia lines which the uninitiated mistake for fossil ferns. On turning it over a miscellaneous collection of invertebrate life —possibly, too, a representative of the higher class in the shape of a spiny agama lizard, or a small snake—is laid bare. Lizards and scorpions seem to favour each other, and live like brothers in peace and amity under the same stone. Under this particular stone lies a yellow and black banded centipede, three little spiders belonging to three different species, a large chrysalis of some veld moth, and two scorpions. The centipede is useless; he has been described years ago, and specimens of his peerage are in every museum. So he may wriggle into the crevices in peace. Two of the spiders also: they are no finds. With the third it is different, for she is a *Solpuga*, and there is just a possibility that Mr. Pocock, of the British Museum, who is specially interested in her ladyship's family, may not have had the pleasure of a previous introduction to her. A bit of stick manipulates her deftly into the open jaws of the methylated jar, where she quickly succumbs. Let there be no regrets over her demise. Full many a Solpuga is born to waste its venom on the desert Karroo, unmourned by any rational creature; but this one perhaps will be enshrined in a white jar on a slip of opaque marble glass with a fine legend below her outspread form, and figure for years in the South Kensington collection. The scorpions are real treasures. This fine creature, so savagely whistling with its tail curled up over its head, ready to dart its sting into the first enemy is *Opisthophthalmus pattisoni*, described by Purcell in the "Annals" some years ago, and a distinct rarity. A magnificent specimen indeed, iridescent violet black over the back of the carapace, and shading off into brown red on the "nippers." It needs a wary hand to catch him. You must wait until he rushes at you, and then nip him deftly with thumb and index at the last tail segment, just where the sting is. The little operation must be smartly executed, else the collector may get stung, and a Karroo scorpion's sting is not to be lightly regarded, although it is not necessarily fatal. As a matter of fact it is easy to execute, and once our friend is grasped in this affectionate manner he is practically helpless. We can lift him up and put him on the palm of the hand—taking care to keep his tail firmly between the fingers—and soothe his savage nature by stroking his back. A big specimen will attempt to bite at our fingers with his mandibles, or nip them with his lobster-like " claws "; but this one is barely three inches in length, and such savagery is hardly likely to abrade the skin. As he lies on the palm we can examine him at leisure, and the study brings out fully his beautiful colouring and excellent shape. Notice the sting at the end of the tail, with the bag of venom attached—a fine, curved little sting, sharp as a hypodermic needle, with a drop of venom exuding from its orifice. By gently squeezing the terminal segment more of this venom may be obtained, and it is easy, from successive captures, to fill a small test tube with this for examination purposes. The stuff is not unlike thin pus in colour and consistency, odourless, acid in reaction, and practically tasteless. It probably contains formic acid—at least in some species—for its application to the bare skin sometimes gives rise to a sharp tingling sensation. When it is injected into the skin in some unfortunate barefooted native boy that has trodden on the animal, it gives rise to a localised swelling, which smarts intolerably. After a few minutes the wound becomes pale, there is a zone of œdema round it, and gradually general symptoms, such as numbness, dryness of the fauces, increased action of the heart, followed by faintness and general pallor come on. In some cases these symptoms may be very severe, leading to a fatal termination in alcoholics or in individuals whose general health is weak. In this respect the seriousness of a scorpion's sting is very similar to that of an adder's bite in England. Quickly treated in an appropriate fashion both accidents are not of grave prognostic significance; but death from scorpion's sting (or " bite," as it is popularly called) is not unknown on the Karroo.

We transfer our capture to the jar, where he soon sinks down—a proof that he has been effectually drowned. A solution of formaldehyde serves as effectually as methylated spirits, and preserves his fine tints even better, but it is an objectionable outfit to carry about on a hot day. The companion scorpion is a small specimen—probably a *Buthus* certainly not belonging to the genus *Opisthophthalmus*—and as there is no time for further investigation, it, too, goes into the jar. There are probably several undescribed species to be discovered yet, especially in the mountains. Under the next stone is a herd of spiders, which can be left severely alone, as none of them are worth capture. The next boulder is entomologically barren, although it yields what the native boy calls a " snake "—a legless lizard. Then comes a likely spot, several flat stones lying together loosely, with Bushman grass growing between them. One yields a fine little red scorpion—a distinct find in this part of the Karroo: underneath the other reposes a viperine, which sails away so rapidly that there is little chance of fixing its specific identity, though the native boy thinks it must be a " Schaaps-steeker." The next adds two different species of Scorpio and Opisthophthalmus to the collecting jar, while the last reveals a burrow. This is merely an irregular, slit-like hole in the clay, but by judiciously excavating with the chisel we lay bare a zig-zaggy tunnel leading into a small cavern, some thirteen inches below the soil level. It is, however, an empty home: there are no scorpions. Farther afield this time underneath a stray Euphorbia bush, is a similar hole, and careful investigation with a stick reveals the fact that there is something in it. With the guiding stick as a director it is easy to lay bare this tunnelled track, and midway we come upon the father of the family himself—a splendid specimen belonging to the same genus as our first friend. He is five inches long and broad in proportion, golden brown with dashes of olive green about the body, and in a tearing rage, wheezing and whistling and attacking the incoming stick like a furious Bombastes. With all his rage he leaves his tail exposed, and it is easy to catch him, although his size and strength make it advisable that he should have no chance of biting or nipping. He is a brand new species—no one has ever described him, so he is a veritable find, and goes into the jar. In six months' time his portrait and history will delight the small section of the scientific world that takes an intelligent interest in his class. Following the track onwards and downwards through the hard clay, we come upon the nest, which is packed with the whole brood. There are some sixteen of them, old and young, all belonging to the same species, and they are transferred, one by one, to the collecting jar.

To the entomologist—using the term generally—scorpions are among the most interesting invertebrates, and a study of their life habits is more absorbing than that of the insects. One or more of our captures can be transferred to the zinc collecting box and brought home alive and in safety, for, notwithstanding all that popular tradition may assert to the contrary, scorpions do not commit suicide. As pets, kept in a sanded cage with sufficient soil to enable them to burrow, their habits can be observed at leisure; but the naturalist who wishes to be on speaking terms with them should study them in their native habitat. There only can he learn to admire their social instincts, their matrimonial and domestic customs, and he will find the study an unusually interesting one. The mere collecting is tyro's work, useful enough for taxonomical purposes, but distasteful to the true naturalist who has learned to classify the various species. Our excuse for the Christmas collecting must be that many of our captures are " nova species," while the others are so rare, notwithstanding the fact that they are common enough in the Karroo, that they are unrepresented in most European museums. Not the least of the pleasures of such a hunt is the opportunities it affords the naturalist of studying the environment of his captures, and of becoming acquainted with their fellow-denizens in the stone colonies. From a medical point of view scorpion venom, too, is a point that needs closer investigation than it has yet received. It is easy to collect in fair quantities, and it is well worthy of study.

